# Bioelectrochemical Properties of Enzyme-Containing Multilayer Polyelectrolyte Microcapsules Modified with Multiwalled Carbon Nanotubes

**DOI:** 10.3390/membranes9040053

**Published:** 2019-04-12

**Authors:** Anatoly Reshetilov, Yulia Plekhanova, Sergei Tarasov, Sergei Tikhonenko, Alexey Dubrovsky, Alexander Kim, Vadim Kashin, Andrey Machulin, Gou-Jen Wang, Vladimir Kolesov, Iren Kuznetsova

**Affiliations:** 1 FSBIS G.K. Skryabin Institute of Biochemistry and Physiology of Microorganisms, Russian Academy of Sciences, Pushchino, Moscow Region 142290, Russia; setar25@gmail.com (S.T.); and.machul@gmail.com (A.M.); 2 FSBIS V.A. Kotelnikov Institute of Radio Engineering and Electronics, Russian Academy of Sciences, Moscow 125009, Russia; vadim_kashin@mail.ru (V.K.); kvv@cplire.ru (V.K.); kuziren@yandex.ru (I.K.); 3 FSBIS Institute of Theoretical and Experimental Biophysics, Russian Academy of Sciences, Pushchino, Moscow Region 142290, Russia; tikhonenkosa@gmail.com (S.T.); Dav198@mail.ru (A.D.); kimerzent@gmail.com (A.K.); 4 Department of Mechanical Engineering, National Chung-Hsing University, Taichung 402, Taiwan; gjwang@dragon.nchu.edu.tw

**Keywords:** multilayer membrane structures, polyelectrolyte microcapsules (PMCs), glucose oxidase, modification with multiwalled carbon nanotubes, PMC impedance decrease

## Abstract

This work investigated changes in the biochemical parameters of multilayer membrane structures, emerging at their modification with multiwalled carbon nanotubes (MWCNTs). The structures were represented by polyelectrolyte microcapsules (PMCs) containing glucose oxidase (GOx). PMCs were made using sodium polystyrene sulfonate (polyanion) and poly(allylamine hydrochloride) (polycation). Three compositions were considered: with MWCNTs incorporated between polyelectrolyte layers; with MWCNTs inserted into the hollow of the microcapsule; and with MWCNTs incorporated simultaneously into the hollow and between polyelectrolyte layers. The impedance spectra showed modifications using MWCNTs to cause a significant decrease in the PMC active resistance from 2560 to 25 kOhm. The cyclic current–voltage curves featured a current rise at modifications of multilayer MWCNT structures. A PMC-based composition was the basis of a receptor element of an amperometric biosensor. The sensitivity of glucose detection by the biosensor was 0.30 and 0.05 μA/mM for PMCs/MWCNTs/GOx and PMCs/GOx compositions, respectively. The biosensor was insensitive to the presence of ethanol or citric acid in the sample. Polyelectrolyte microcapsules based on a multilayer membrane incorporating the enzyme and MWCNTs can be efficient in developing biosensors and microbial fuel cells.

## 1. Introduction

Recent research has witnessed an increased interest in developments of micro- and nanostructures to be used in miniature devices [1]. Microstructures can be developed based on various materials, some of which are frequently used polyelectrolytes [2,3]. Polyelectrolyte microcapsules (PMCs), fabricated by alternate layering of oppositely charged polyelectrolytes to nano- and microsize disperse particles, are subjects of a rapidly developing field of polymer nanotechnology [4,5,6]. The approach has been first described by Decher and coworkers [7,8]. On the whole, polyelectrolyte multilayers/multilayer membranes are widely used in many applications, including biosensors [9,10,11], asymmetric membranes for gas separation [12], and tissue engineering [13]; they have also been used for antibacterial purposes [14,15].

The PMC multilayer membrane has a protective action on objects inside the capsule, this enables using microcapsules as microcontainers and microreactors in medical [16], light [17,18], and agricultural industries [19]. Enzymes immobilized into polyelectrolyte microcapsules can be used to develop biosensors, e.g., for assaying urea [20,21,22], acetylcholine [23], and glucose [24].

When forming biosensors and microbial fuel cells (MFCs), an important electrical parameter to be considered is the conductance of measuring electrode material. One of the modern tendencies in choosing electrode materials for MFCs and biosensors is, at present, the use of nanostructured materials for modifying electrodes, which is due to their unique physical and chemical properties [25]. It is known that the modification of the measuring electrode with carbon nanomaterials results in the decrease of its resistance, as well as in the increase of the active surface of the working electrode [26]. The most promising materials for developing bioelectrodes in bioelectrochemistry are carbon nanotubes [27], graphene [28], mesoporous carbon [29], and carbon black [30], owing to their high porosity, surface area, and conductance [31].

The bulk of works on the modification of polyelectrolyte layers with nanomaterials is related to the development of biosensors based on glassy carbon electrodes [32]. In several works [33,34], a composition of glucose oxidase (GOx) and multiwalled carbon nanotubes (MWCNTs) was immobilized into layers of osmium polymers and used as the base for screen-printed amperometric glucose biosensors. Those biosensors demonstrated a good stability and low detection limit but suffered from hindered glucose transport through the multilayers of osmium polymers. As far as we are aware, modifications with carbon nanotubes of closed multilayer membrane structures represented by enzyme-containing PMCs have not been carried out.

The aim of this work was to investigate changes in bioelectrochemical parameters of polyelectrolyte microcapsules formed from multilayer membranes containing the enzyme (GOx) at their modification with multilayer carbon nanotubes.

## 2. Materials and Methods

### 2.1. Reagents

Reagents used: sodium chloride, dipotassium hydrogen phosphate trihydrate, sodium hydroxide, glucose, and acetic acid (Diakon, Russia); iron(III) chloride, potassium chloride, calcium chloride, and sodium carbonate (Khimmed, Russia); hydrochloric acid, hydrogen peroxide (30% solution), sodium carbonate, and potassium hexacyanoferrate(III) (HCF) (Reakhim, Russia); chitosan (low molecular weight), sodium polystyrene sulfonate (PSS, 70 kDa), poly(allylamine hydrochloride) (PAH, 70 kDa), ethylenediaminetetraacetic acid (EDTA), and 4-aminoantipyrine, phenol, glucose oxidase (EC 1.1.3.4) from *Aspergillus niger* (activity, 185000 U/g) (Sigma-Aldrich, USA). Taunit-M multiwalled carbon nanotubes (NanoTechCentre LLC, Tambov, Russia) were used for modification of the electrode.

Screen-printed three-contact electrodes (Color Electronics, Moscow, Russia) containing Electrodag 6017SS graphite paste (Henkel, Germany) were used.

### 2.2. Formation of a Prussian Blue-Based Chemical Sensor

Prussian blue was precipitated on the electrode surface from the reaction mixture containing 0.1 M FeCl_3_ and 0.1 M K_3_ [Fe(CN)_6_] in the background electrolyte (0.1 M KCl, 0.1 M HCl). The mixture was preliminarily kept for 20 min in an Eppendorf test tube in the dark; then it was applied onto the working electrode. After 15 min, hydrogen peroxide was added into the applied solution to a concentration of 100 mM in a drop; the produced mixture was held on the electrode for 45 min. Upon precipitation, the electrode surface was washed with Milli-Q water. After that the electrode was activated (a potentiodynamic treatment of the Prussian blue electrode in a cyclic mode in the background electrolyte from −0.05 up to 0.35 V) in accordance with the method described by Karyakin and co-authors [35].

In special experiments, the surface of the graphite electrode was modified with MWCNTs prior to the precipitation of Prussian blue. For this, a suspension of nanotubes (5 mg/mL) was applied onto the electrode surface and dried for 1 h at 22 °C.

### 2.3. Formation of CaCO_3_-Protein Microspherolites

For the preparation of microcapsules, an equal volume of a sodium carbonate solution (0.33 M) was added to a 0.33 M calcium chloride solution and intensively stirred on a magnetic mixer. The mixing was continued for 30 s, after which the suspension was kept up to the complete sedimentation of formed particles [36]. Maturation of microspherolites was monitored by a light microscope. Then the solution was filtered from the supernatant, the sediment was washed with distilled water and used to produce PMCs.

For the preparation of microcapsules containing GOx, an equal volume of a sodium carbonate solution (0.33 M) was added to a 0.33 M calcium chloride solution containing 3 mg/mL GOx and intensively stirred on a magnetic mixer.

To produce microcapsules containing MWCNTs in the hollow, a suspension of nanotubes was mixed with a solution of CaCl_2_.

To produce microcapsules containing MWCNTs in the hollow and GOx simultaneously, a suspension of nanotubes was mixed with a solution of Na_2_CO_3_, and an enzyme solution was mixed with a solution of CaCl_2_. Further on, from these mixtures we formed composite microspherolites of various structures (CaCO_3_; CaCO_3_/GOx; CaCO_3_/MWCNTs; CaCO_3_/GOx/MWCNTs) by the above-described technique.

### 2.4. Preparation of PMCs

Polyelectrolyte microcapsules were obtained by alternate adsorption of oppositely charged polyelectrolytes on disperse microparticles (cores) with subsequent dissolution of the cores. Alternate adsorption of PAH and PSS on the surfaces of CaCO_3_ microspherolites was carried out in polyelectrolyte solutions (2 mg/ml) containing 0.5 M NaCl. Each adsorption step was followed by a triple washing of fabricated capsules with a 0.5 M NaCl solution, which was required to discard nonadsorbed polymer molecules. The supernatant was centrifuged to separate the particles. After the required number of layers was deposited, carbonate cores were dissolved for 12 h in a 0.2 M solution of EDTA. Polyelectrolyte capsules were rinsed three times with Milli-Q to remove cores’ components. Capsules contained six polyelectrolyte layers because capsules with fewer layers were unstable. The suspension of capsules was stored in bidistilled water at a temperature of 4 °С.

For the preparation of PMCs/MWCNTs between layers of the polyelectrolytes, microspherolites with two layers of PAH and one layer of PSS were put into a suspension of nanotubes. After 5 min, the microspherolites were washed three times in a 0.5 M NaCl solution, then the remaining three layers of PAH and PSS were added.

**Figure 1:**
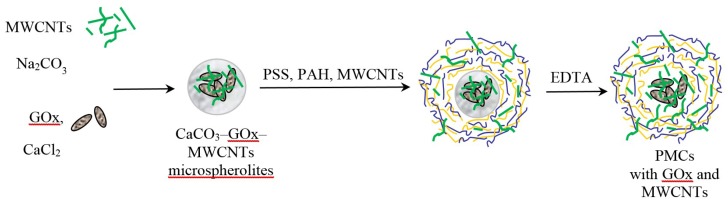
Formation of polyelectrolyte microcapsules with the enzyme and nanotubes.

Formation of PMCs around the enzyme is shown schematically in Figure 1.

### 2.5. Formation of Glucose Biosensors

Polyelectrolyte microcapsules, in the amount of 5 μL with incorporated GOx, were applied onto the surface of a Prussian blue electrode and dried at 22 °С for 30 min. Between measurements, the biosensors were stored at a temperature of 4 °С in the dark. The measurements were carried out at a temperature of 22 °С in a 1-ml cell at constant stirring. The measurements were performed on an IPC-Micro galvanopotentiostat (Kronas Ltd, Russia). Glucose solutions were prepared in a 25 mM sodium–potassium–phosphate buffer solution (pH 6.5) with the addition of 20 mM NaCl.

The voltammetric and impedance measurements were carried out with a VersaSTAT 4 potentiostat galvanostat (Ametek Inc., USA) in the same solution with the addition of 5 mM [Fe(CN)_6_]_3_. A scanning rate of 40 mV/s was used for voltammetric measurements. A 100 mV constant potential (40 kHz–0.02 Hz frequency range) and a voltage modulation of 10 mV were used to obtain impedance spectra. The correct equivalent circuit for every system was picked using ZSimpWin software (EChem Software, USA). The measurements were carried out with constant stirring of solutions.

### 2.6. Optical Microscopy

The optical microscopy examination of PMCs was conducted in the phase contrast mode using a Nikon Eclipse Ci microscope (Nikon, Japan) with an image registration camera ProgRes SpeedXT core5 (Jenoptik, Germany). The size of microcapsules was calculated from the average measured sizes of 100 capsules.

### 2.7. Scanning Electron Microscopy

PMCs were dehydrated in a series of alcohols of increasing concentrations (from 50 up to 100%) for 20 min at each stage. Then they were suspended in tert-butanol (Sigma-Aldrich) two times for 20 min each at 26 °C. Further on, the PMCs were held in tert-butanol for 12 h at 4 °C. The specimens were then freeze-dried (JFD-320, JEOL, Japan). An adhesive tape was used for the preparation of open capsules.

The dried specimens of both types were fixed on aluminium disks by means of a current-conducting tape and coated with gold using a vacuum sputtering equipment JFC-1100 (JEOL, Japan) for better resolution. The specimens were examined in a JSM-6510LV scanning electron microscope (JEOL, Japan).

### 2.8. Atomic Force Microscopy

Freshly cleaved supports from highly oriented pyrolytic graphite (HOPG) were used to investigate PMCs by atomic force microscopy (AFM) methods. The upper layers of HOPG were cleaved directly in a PMC-containing solution. The studies were carried out by a Solver P47 H scanning probe microscope (NT-MDT, Russia) in the semicontact mode in air using a diamond cantilever.

## 3. Results

### 3.1. Characterization of PMCs by AFM and SEM

**Figure 2:**
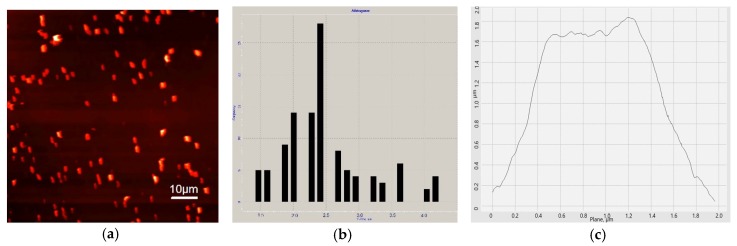
Atomic force microscopy (AFM) images of microcapsules. An AFM micrograph of a layer of aggregated and single polyelectrolyte microcapsules (PMCs) (**a**); a histogram of 100 microcapsules (**b**); and the geometric size of a single microcapsule (**c**).

Micrographs of structures that characterize the composite-electrode material are shown in Figure 2. Figure 2a presents a PMC layer applied to the freshly cleaved surface of HOPG, where both single and aggregated PMCs are seen. The shape and profile of a single microcapsule are shown in Figure 2b,c, respectively. The size of a single PMC was of the order of 2.5 ± 0.6 μm (based on 30 measurements).

**Figure 3:**
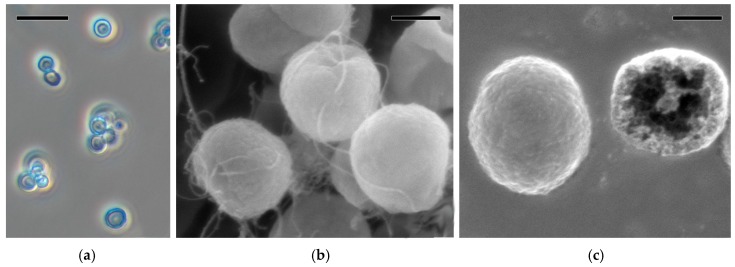
PMC micrographs obtained by phase-contrast microscopy (**a**) and scanning electron microscopy ((**b**) intact capsules and (**c**) open capsules). Scale bars are 10 μm (a) or 1 μm (**b**,**c**).

PMC micrographs obtained by phase-contrast and scanning electron microscopies are shown in Figure 3.

The size of a single microcapsule was of the order of 2.8 ± 0.5 μm (based on 50 measurements). This estimate coincides with that for a single microcapsule shown in Figure 2b. It is seen in Figure 3a that a minor conjugation of PMCs takes place. Figure 3b,c gives an idea of the PMC structure. The thickness of the multilayer membrane consisting of six layers of oppositely charged polyelectrolytes was 37 ± 3 nm (each layer was ~6 nm) as described by Kazakova and co-authors [37]. Noteworthy is the spatial arrangement of MWCNT threads. Figure 3c shows a case when MWCNTs were incorporated simultaneously between layers of polyelectrolytes and into the hollow of a microcapsule. MWCNT threads partially protruding to the PMC surface are seen.

### 3.2. Impedance Characteristics of Various Types of Electrodes

**Figure 4:**
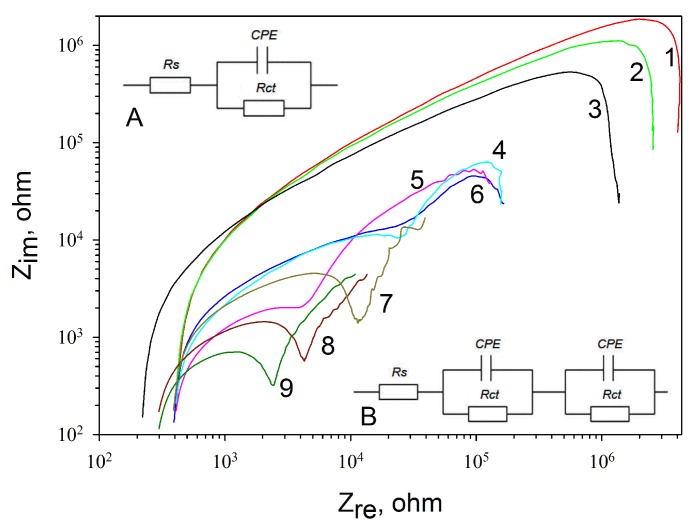
Impedance spectra for electrodes with various surface modifications: 1, a nonmodified electrode; 2, with PMCs; 3, glucose oxidase (GOx); 4, PMCs containing multiwalled carbon nanotubes (MWCNTs) in the hollow and between polyelectrolyte layers; 5, PMCs containing MWCNTs between polyelectrolyte layers; 6, PMCs containing MWCNTs in the hollow; 7, PMCs with GOx; 8, MWCNTs; and 9, PMCs with GOx and MWCNTs in the hollow. Inset shows the equivalent electric circuits used for electrodes without MWCNTs (A) and for electrodes with MWCNTs (B).

The impedance of the electrode depends on a multitude of factors; herewith, it changes at the application of any substance onto the electrode surface. Measurements were carried out using electrochemical impedance spectroscopy by the three-electrode scheme in the presence of 5 mM hexacyanoferrate at an applied potential of 100 mV. The composition of the solution remained invariable, so any changes in the impedance spectra were related only to the modification of the electrode surface. The impedance spectra for the control states (a nonmodified screen-printed electrode without any coating or electrodes with a coating of PMCs, GOx, PMCs/GOx, and MWCNTs) and MWCNT-modified (PMCs/MWCNTs in the hollow of the PMCs and between polyelectrolyte layers, PMCs containing MWCNTs between polyelectrolyte layers, PMCs containing MWCNTs in the hollow, PMCs containing GOx, and MWCNTs in the hollow) are presented in Figure 4. For convenience of comparison, the frequency impedance curves are given in logarithmic coordinates. The impedance spectra were processed using a Randles modified scheme with a constant phase element (CPE) replacing the capacitance of the electrical layer (Figure 4, Inset A). It should be noted that the typical form of the frequency impedance curves changed when a PMC layer was present on the surface of the electrode. We observed two semicircles, each of which featured a separate pair of CPEs and a charge-transfer resistance *R*_ct_, instead of one semicircle on the frequency curve (Figure 4, Inset B).

The values of active resistances obtained for each modification of the electrode are given in Table 1. The highest resistance values were obtained for screen-printed electrodes without applying any coatings, as well as for electrodes modified separately by the enzyme and PMCs. The lowest value of the resistance was observed for a composition including PMCs, GOx, and MWCNTs in the hollow and between polyelectrolyte layers. Herewith, the resistance was found to decrease from 120 to 25 kOhm as compared with the composition with PMCs/GOx without MWCNTs. Thus, it can be concluded that the incorporation of MWCNTs into PMCs contributes to a better electron transfer in the system, which, in turn, has a positive effect on the characteristics of biosensors based on PMCs/GOx/MWCNTs.

### 3.3. Cyclic Voltammograms of Modified Electrodes

**Figure 5:**
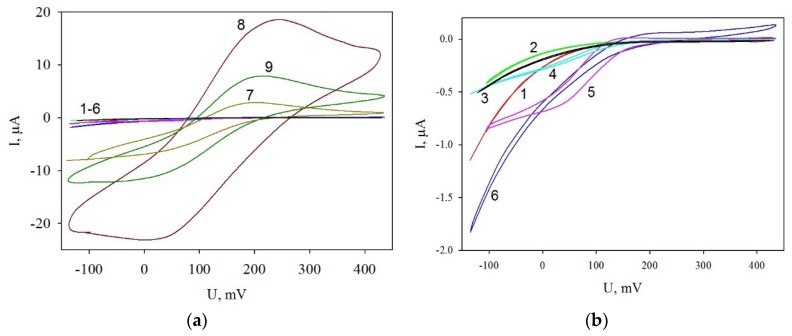
Cyclic voltammograms of modified electrodes: 1, a nonmodified electrode; 2, with PMCs, 3, GOx; 4, PMCs containing MWCNTs in the hollow and between polyelectrolyte layers; 5, PMCs containing MWCNTs between polyelectrolyte layers; 6, PMCs containing MWCNTs in the hollow; 7, PMCs with GOx; 8, MWCNTs; and 9, PMCs with GOx and MWCNTs in the hollow. Measurements were carried out with the addition of 5 mM HCF. For comparison, **a** shows all types of dependences, while **b** represents the zoom of curves 1–6.

For each modification of the electrode surface cyclic voltammograms were recorded (Figure 5). As the recordings show (Figure 5b, curves 1–6), modifications of the graphite electrode with PMCs at an addition of MWCNTs, both in the hollow and between polyelectrolyte layers, has no significant effect on the cyclic voltammogram shapes. Modification of PMCs with the enzyme has no effect on the voltammograms either. Their shape changes significantly at a modification of the electrode surface with MWCNTs (Figure 5a, curve 8). The changes are due to the emergence of characteristic redox peaks. The values of the peaks decrease significantly at the modification of the electrode surface with PMCs with the enzyme (curve 7); they rise again—but fail to reach the initial value—at addition of microcapsules with enzyme-containing MWCNTs into the hollow (curve 9). Thus, the electrochemical measurements prove a decrease of resistance in the system at its modification with nanotubes. This is supported by both the impedance data and the cyclic voltammograms of the considered electrodes.

Microcapsules with the enzyme were immobilized by sorption on the graphite-electrode surface. The upper layer of polyelectrolyte covering the capsule carries a negative charge and sorbs well on the surface of the graphite electrode owing to Coulomb interaction. To investigate the characteristics of glucose biosensors, the electrode surface was preliminarily modified with Prussian blue. Prussian blue is an electrocatalyst of the reduction of hydrogen peroxide [38,39], which evolves during the oxidation of substrate (glucose) by the enzyme and reflects the catalytic activity of the immobilized enzyme [35].

### 3.4. Determination of Kinetic Parameters of PMC/GOx and PMC/GOx/MWCNTs Biosensors

**Figure 6:**
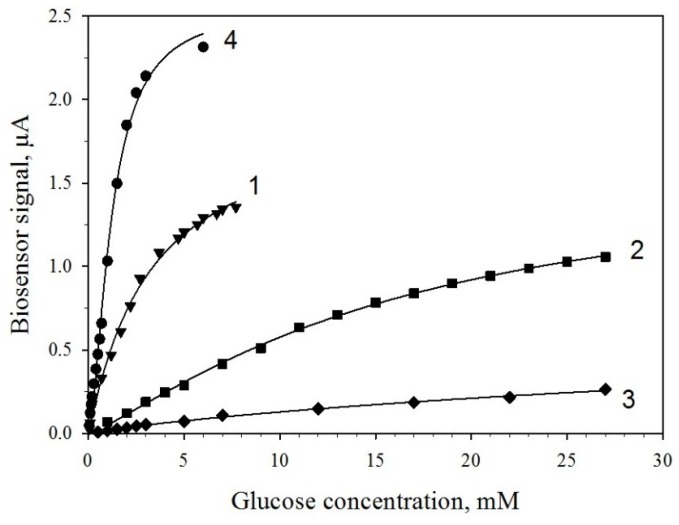
Calibration dependences of biosensor signals vs. glucose concentrations at various modifications of the electrode: 1, PMCs/GOx/MWCNTs in the hollow; 2, PMCs/GOx; 3, GOx; and 4, PMCs/GOx/MWCNTs in the hollow plus an additional modification of the electrode with MWCNTs (see explanations in the text).

We investigated the characteristics of biosensors based on electrodes with Prussian blue, modified with enzyme-containing PMCs. Capsules with and without MWCNTs in their composition were used. For comparison, we used GOx immobilized by simple sorption on the surface of the electrode. Figure 6 shows the obtained calibration dependences for the biosensors, Table 2 presents the numerical values of the main characteristics. Introduction of MWCNTs into the capsules leads to higher sensitivity of the biosensor to glucose (0.30 μA/mM vs. 0.05 μA/mM) at the same concentration of the enzyme on the electrode (Figure 6, curves 2 and 3). Additional modification of the electrode surface with nanotubes leads to a further increase of biosensor signals (Figure 6, curve 4) and a significant (up to 0.94 μA/mM) assay sensitivity increase. This is due to an increase of the conductivity of the system and a possible facilitated transfer of electrons from the nanotubes to the electrode, which is also supported by the data of impedance measurements. Besides, a modification of the electrode with MWCNTs changes the glucose detection range (Table 2). The lower range of detection is shifted towards lower concentrations, which are 0.05 mM, which enables assaying lower concentrations of glucose. Table 3 presents an overview of GOx-based biosensors for glucose detection. The attained results, coupled with the relative simplicity of the biosensor construction, compare favorably with previously reported biosensors. No study regarding glucose quantification based on PMC/GOx/MWCNTs s-based biosensors was found.

**Figure 7:**
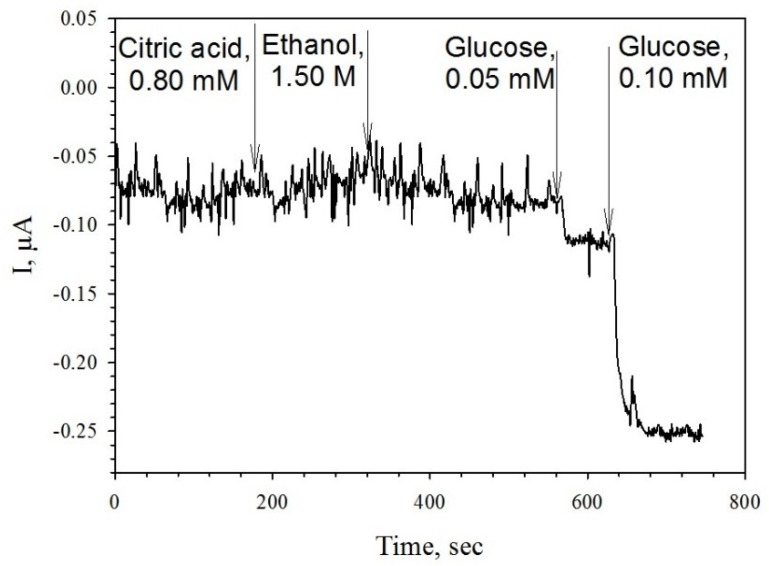
Amperometric signals of a PMCs/GOx/MWCNTs-modified biosensor upon addition of citric acid, ethanol, or glucose.

One of the important characteristics of the assay is its specificity. To assess a partial specificity of the receptor element for a biosensor based on MWCNTs-modified electrodes, we examined signals for citric acid (within the concentration range of 0.06 to 0.90 mM) and ethanol (within the concentration range of 0.1 to 2.0 M). As shown in Figure 7, these compounds evoked no generation of useful signals. This is due, first, to the specificity of the enzyme we used, and, second, to the fact that the measurements were carried out at a zero potential of the measuring electrode.

## 4. Discussion

The performed work aimed to study effects of modulating the bioelectrochemical properties of PMCs at their contact with MWCNTs. Polyelectrolyte microcapsules are objects of a rapidly developing field—polymer nanotechnology—and are widely used to form supramolecular structures. PMCs are, in fact, a structure formed by multilayer membranes that consist of polyelectrolytes.

PMCs are widely used in experimental and applied events. They make it possible to create containers for incapsulation of proteins, including enzymes, (sub)cellular structures, medicinal substances, and other high-molecular compounds. The incorporation of enzymes into PMCs leads to a significant rise in the lifetime of its active state. In this connection, it is topical to investigate their properties at their contact with a nanomaterial represented by MWCNTs.

This work was the first to show that such characteristics as impedance spectra of PMCs prove to be highly sensitive to the occurrence of MWCNTs. This sensitivity manifests itself by a significant decrease of the active constituent of the impedance at a modification with PMCs. On the whole, effects of a change in the physicochemical properties of multilayer polyelectrolyte membranes have been known for various electrode materials [46], including for modifications of multilayer polyelectrolyte membranes using MWCNTs, which make the base of the biosensor [32]. It should be noted that in earlier considered cases, the modification with MWCNTs was considered either for plane multilayer membranes or else for membranes formed from other classes of polyelectrolytes, e.g., osmium polymer [33] or chitosan [47]. The presented work first shows that, using MWCNTs, a significant change of the physical chemical properties of closed membrane structures can be obtained. It is shown that the decrease in the resistance of PMCs due to the inclusion of MWCNTs in its composition leads to an increase in the sensitivity of glucose biosensors and a decrease in their detection limit. The produced data of impedance measurements were supported by a study of the cyclic current–voltage dependences. Studies of a laboratory model of the biosensor enabled in the first approximation to assess the analytical parameters, which indicate a significant improvement of the characteristics.

We should note an important applied advantage that ensues from the preparation of closed microstructures as compared with the formation of plane structures. Closed cell-like microstructures act as a semiproduct, which enables creating a required product, e.g., a receptor element of the biosensor, from the available stock with minimal time expenses. Technology-wise, the development of such kinds of items from plane structures appears to be impossible. For this reason, separately fabricating and storing PMC capsules containing a biocatalyst and MWCNTs and then rapidly using them is a way of forming biosensors or else microbial fuel cell structures over small time periods, and does not require additional chemical reagents to treat the electrode surface before and after the measurement. As our research has shown, the obtained electrode composite demonstrates good reproducibility of voltammetric responses, as well as a low detection limit and high sensitivity for measuring glucose. Moreover, GOx is a suitable model biocatalyst for the further development of other enzyme bioelectrodes containing PMCs and nanomaterials. Composites presented in this paper may be used as membranes, whose properties can be controlled during production.

## Figures and Tables

**Table 1:** Active resistances of screen-printed electrodes with various modifications of the surface.

Graphite Electrodes Modified with Various Components	Total Resistance, kOhm
Nonmodified screen-printed electrodes	4200 ± 200
PMCs	2560 ± 170
PMCs/MWCNTs in the hollow	172 ± 8
PMCs/MWCNTs between layers of polyelectrolytes	181 ± 6
PMCs/MWCNTs in the hollow and between layers of polyelectrolytes	162 ± 6
GOx	1200 ± 30
MWCNTs	34 ± 1
PMCs with GOx	120 ± 4
PMCs with GOx and MWCNTs in the hollow and between layers of polyelectrolytes	25 ± 1

Note: the mean values of five measurements and a mean root square deviation for the total active resistances are given.

**Table 2:** Basic analytical characteristics of biosensors.

	Composition of Biocatalyst	PMCs/GOx/MWCNTs *	PMCs/GOx/MWCNTs	PMCs/GOx	GOx
Parameter					
Equation describing the calibration dependence		$V=\frac{V_{\max}S^{h}}{K_{M}^{h}+S^{h}}$			
Parameter values of the calibration dependence		*V*_max_ = 2.560; *h* = 1.667; *K*_m_= 1.203; *R*^2^ = 0.99	*V*_max_ = 1.938; *h* = 1.050; *K*_m_ = 3.194; *R*^2^ = 0.99	*V*_max_ = 1.639; *h* = 1.238; *K*_m_ = 16.393; *R*^2^ = 0.99	*V*_max_ = 0.959; *h* = 0.865; *K*_m_ = 86.692; *R*^2^ = 0.99
Linear range of detection, mM		0.05–2	0.2–2.7	1–15	0.5–7
Regression equation for the linear segment, correlation coefficient *R*^2^		*y* = 0.9443*x* + 0.0193, *R*^2^ = 0.99	*y* = 0.2966*x* − 0.1161, *R*^2^ = 0.99	*y* = 0.0524*x* + 0.0293, *R*^2^ = 0.99	*y* = 0.0149*x* + 0.0029, *R*^2^ = 0.99
Sensitivity coefficient, μA/mM		0.94	0.30	0.05	0.01
Minimal range of detection, mM		0.05	0.05	1	0.5
Detection range, mM		0.05–3	0.05–6	1–25	0.5–25

* For this type of electrode the surface was additionally modified with MWCNTs (see methods).

**Table 3:** Sensitivity and limit of detection for some glucose biosensors based on GOx.

Biosensor Composition	Limit of Detection, μM	Sensivity	References
Nafion/GOx/ZnO/ITO	50	3.87 µA/mM·cm^2^	[40]
PEC/AuNPs/GOx/Au	5	283.9 µA/log[glucose]	[41]
Poly(3,4-ethylenedioxythiophene) nanofiber based glucose biosensor	67.8	272.58 μA/mM cm^2^	[42]
GOx/AgNPs/HNTs	200	5.1 μA/mM cm^2^	[43]
rGO/PDA/MOF/GOx	0.3	9.6 μA/mM cm^2^	[44]
p-MAA/Nafion/GOx	10	12.0 μA/mM cm^2^	[45]
PMCs/MWCNTs/GOx	50	13.4 μA/mM cm^2^	This work

Note: ITO, indium tin oxide; PEC, polyelectrolyte complex; NP, nanoparticles; HNTs, halloysite nanotubes; rGO, reduced graphene oxide; PDA, polydopamine; MOF, metal-organic frameworks; p-mAA, poly-methacrylic acid.
